# Spatio-temporal distribution and acoustic characterization of haddock (*Melanogrammus aeglefinus*, Gadidae) calls in the Arctic fjord Kongsfjorden (Svalbard Islands)

**DOI:** 10.1038/s41598-020-75415-9

**Published:** 2020-10-26

**Authors:** G. Buscaino, M. Picciulin, D. E. Canale, E. Papale, M. Ceraulo, R. Grammauta, S. Mazzola

**Affiliations:** 1 Bioacousticslab, National Research Council, via del Mare, 6, Torretta Granitola, Campobello di Mazara, TP Italy; 2 Ca’ Foscari University of Venice, Venezia Mestre, Italy

**Keywords:** Behavioural ecology, Behavioural ecology

## Abstract

In this study we analysed the acoustic properties and presence of haddock calls in the Arctic fjord Kongsfjorden (79° N–12° E, Svalbard Islands, Norway) in one year. Data were collected with three autonomous acoustic recorders located in the inner, middle, and outer parts of the fjord. The fjord is characterized by a gradient of oceanographic conditions from the inner to the outer part, reflecting changes from Arctic to Atlantic waters. Haddock sounds were more abundant in the outer fjord than in the middle fjord, whereas they were absent at the inner site. Mainly at the open-water site, the call abundance exhibited strong periodicity and a correlation with the cycles of neap tide (15 days) in August, with a clear diel cycle (24 h) in September and October. This result suggests that in this extreme environment with 24 h of light during summer, haddock regulate their acoustic activity according to the main available oscillating external physical driver, such as tide during the polar summer, while when the alternation of light/dark starts, they shift the periodicity of their calls to a diel cycle. Calls were recorded outside the spawning period (from July to October), and their characteristics indicated non-reproductive communicative contests. By using a detailed sound analysis based on previous laboratory studies for the first time, we suggest that the monitored population contains mainly juveniles (44% compared to 41% females and only approximately 15% mature males), showing the predominance of females in the middle fjord and juveniles at the open-water site.

## Introduction

The haddock (*Melanogrammus aeglefinus*) is a widely distributed fish belonging to the Gadidae family and represents an important target species for the fishery industry. It lives in the Atlantic and in the Barents Sea at a latitudinal range between 38° and 80° N, usually at depths between 80 and 200 m and at temperatures between 2 and 10 °C^1^. Haddock aggregate during the spawning period, i.e., in March to April in the deepest parts of the North Sea^2^ and up to early June off the coast of Norway^3^. Little is known about the habitat where haddock spawning occurs and which features promote fish aggregations. Usually spawning fish aggregate close to the seabed^4^ at depths of 400–500 m off the coast of Norway, but also in shallower waters in Vestfjord^5^ or in Balsfjord^6^. Reproductive maturity is reached at 4 years at a length of 40–60 cm^1^.

Similar to other gadoid species^7^, the haddock is a vocal species; its sounds consist of a series of pulses (knocks) composed of two short pulses at low frequencies^8^. Sound production varies throughout the year, but it peaks during the reproductive season^9^, when the haddock produces a diversity of calls associated with particular behavioural contexts^8^. Studies performed in aquarium tanks revealed that the sounds are mostly produced by males during patrolling displays, which are both a territorial and a spawning behaviour in haddock^9^. Interindividual differences in the knock produced by patrolling males have been demonstrated, likely reflecting changes in the drumming muscles involved in sound production. As courtship proceeds, knocks are repeated at a faster rate culminating in a continuous hum^8,10^ and eventually leading to spawning. Outside the spawning season, *M. aeglefinus* calls are slightly different, composed mostly of two or more pulses of longer duration^11^.

Sounds are also produced by females and by juveniles. Casaretto et al.^12^ showed that the structure of the haddock sound unit changes according to fish maturity and is sex-specific. These differences are explained by sexual dimorphism in the generating mechanism, with drumming muscle size varying according to sex, age, maturity and season^12,13^.

Haddock vocalizations recorded in captivity have been used for the identification of this species in the sea. At the head of the Balsfjord (Norway), reproductive haddock aggregations have been found by means of their vocalizations in several locations^6^. The majority of the recorded sounds were very similar to those produced by patrolling males in the aquarium, and the presence of sounds with a fast repetition rate indicated that the fish were close to spawning. This confirmed the use of passive acoustic monitoring for studying the biology and distribution of the species. Neverthless, we are not aware of any published long-term studies on vocalizing Gadidae fish species in the sea. In addition, although captivity studies suggest the possibility of using haddock sound structure to infer the sex and maturity of emitters, similar work has not been applied to sea recordings.

Long-term studies investigating temporal patterns of fish calls are available for low-latitude geographical areas, such as the Mediterranean Sea^14,15^, Georgia^16^, USA waters^17^, the Gulf of Mexico^18^, Australian waters^19^, and in Argentinean waters^20^. In these studies, it was commonly demonstrated that sound activity increases during the reproductive season, with vocal peaks during nighttime hours. Photoperiod and temperature are the principal natural cues triggering seasonal maturation and acoustic activity in fish, as observed infamily Scenidae^18,21^. In polar environments, however, the strength of the daily light–dark cycle is greatly reduced during summer, when there is almost continuous daylight. Few studies have investigated the presence of behavioural rhythmicity in the absence of light–dark alternation^22,23^, but variable behavioural responses to continuous polar lighting have been highlighted. In the small polar cod (*Boreogadus saida*), for example, diel vertical migration is exactly synchronized with the changing of day and night, and when the midnight sun starts in late May, this migration stops^24^; however, in another gadoid species, the burbot (*Lota lota*), the activity rhythms persist during the polar day or night^25,26^. Northward expansion of boreal Atlantic cod (*Gadus morhua*) and haddock (*Melanogrammus aeglefinus*) (in particular juvenile fish) into areas inhabited by the native polar cod (*Boreogadus saida*) has been observed only in the recent period of increased inflow of warmer Atlantic water to the western coast of Svalbard^27,28^, and no data on their rhythmicity have been reported. Thus far, seasonal patterns of haddock calls have been studied only in tanks with an alternating photoperiod^9^.

In the Svalbard Archipelago (Norway), the haddock represents an important species for the commercial fishery, with more than 239,000 tons caught between 1980 and 2013^29^, making it the third most important fish species captured after the capelin (*Mallotus villosus*) and the cod (*Gadus morhua*). In recent years, an increase in haddock captures was recorded. This can probably be partially attributed to a northward shift of some demersal species as waters have warmed, the sea ice extent has decreased^30^ and larger feeding areas have become available during the light season^27,29^.

The main aims of this study are to (1) characterize haddock calls in the Arctic environment, (2) to evaluate the spatio-temporal distribution of the haddock by using its calls, (3) explore the correlations between haddock acoustic activity and external physical factors such as tide and solar elevation and (4) discuss the use of acoustic data for detecting fish age classes and sex and haddock behavioural patterns.

## Results

A total of 7751 h of recordings from three sites that covered a period of one year (from April 2014 to March 2015) were analysed (see Table 1). We did not record any haddock sounds at the glacier site, whereas sounds attributed to haddocks were recorded at the other two sites.

**Table 1:** Recording sites spatial locatization and sampling effort for every month.

	Site					
	Open-water (79.032° N–11.546° E) Depth, m 75		Middle-fjord (78.937° N–11.91° E) Depth, m 20		Glacier (78.912° N–11.404° E) Depth, m 75	
	Recording hours	Sampled days	Recording hours	Sampled days	Recording hours	Sampled days
April	135	11	199	17	135	11
May	359	30	230	19	360	30
June	359	30	256	21	359	30
July	334	28	254	21	310	26
August	372	31	372	31	370	31
September	360	30	360	30	360	30
October	116	10	188	16	190	16
November	120	10	120	10	120	10
December	121	10	95	8	120	10
January	132	11	132	11	132	11
February	108	9	108	9	108	9
March	121	10	121	10	120	10
Total	2635	220	2433	203	2683	224
Yearly duty cycle, %	30	60	28	56	31	61

### Temporal patterns of haddock vocal activity and their relations with solar elevation and tide

**Figure 1:**
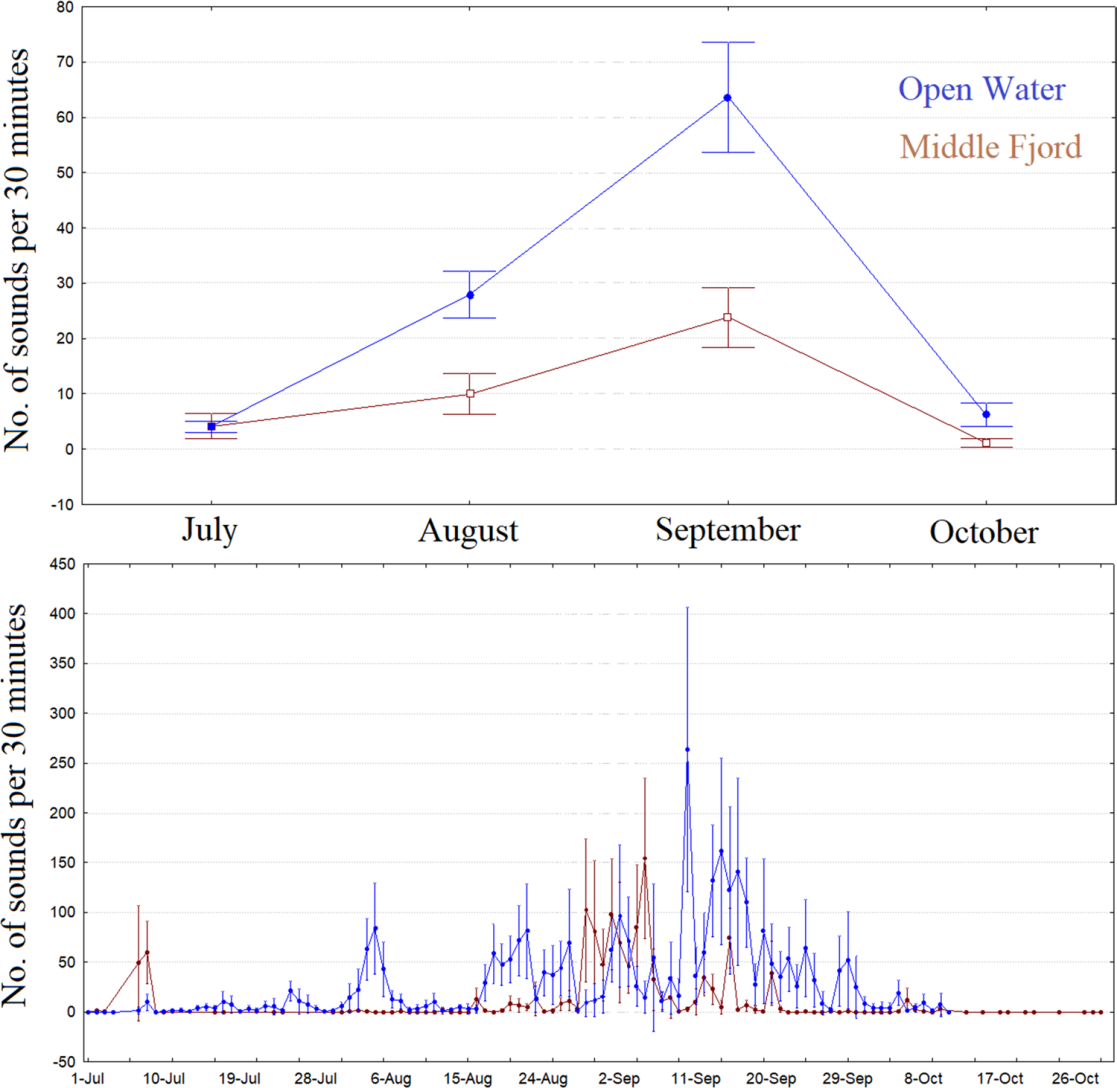
Upper: Mean (± standard error) number of haddock sounds in 30 min for each month. Lower: Mean (± standard error) number of haddock sounds in 30 min on each day of sampling; only months in which haddock sounds were recorded are presented (July–October). The glacier site is not shown because no haddock sounds were detected.

Haddock sounds at both the middle and open fjord sites were first recorded from July until October (Fig. 1). No haddock sounds were recorded during the known spawning period of the species (March–April). Significant differences in the abundance of sounds were found between the middle-fjord and open-water sites when considering the whole period of acoustic activity, i.e., from July to October (Mann–Whitney U test; p < 0.0001). We detected significant differences in acoustic activity among the sites in all months except July (Fig. 1). The site where the largest number of sounds were detected was the open-water site (see Fig. 1), with a peak of 264 ± 68 (mean ± standard error) calls per 30 min found on 11 September 2014. At the middle-fjord site, we recorded a peak on 5 September 2014, with 154 ± 39 (mean ± standard error) calls per 30 min.

**Figure 2:**
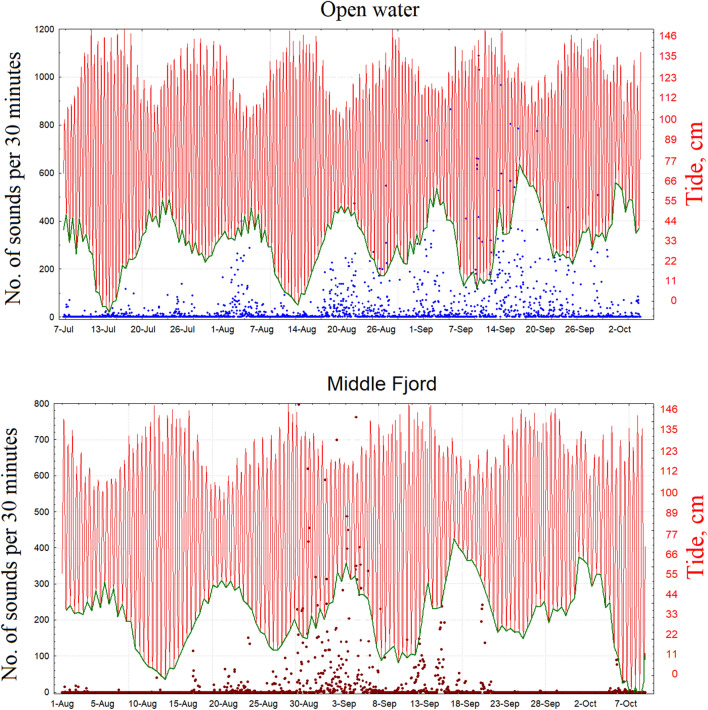
Counts of haddock sounds in each 30 min file during the acoustic activity period for the two sites (blue and red points for the open-water and middle-fjord sites, respectively). The tide (red line) and the DE-Tide (green line) are also shown.

The counts of haddock sounds for each 30 min file during the acoustic activity period for the two sites are reported in Fig. 2 (see blue and red points for the open-water and middle-fjord sites, respectively). In the same figure, the tide (red line) and the neap tide (DE-Tide) (green line) are also shown.

**Figure 3:**
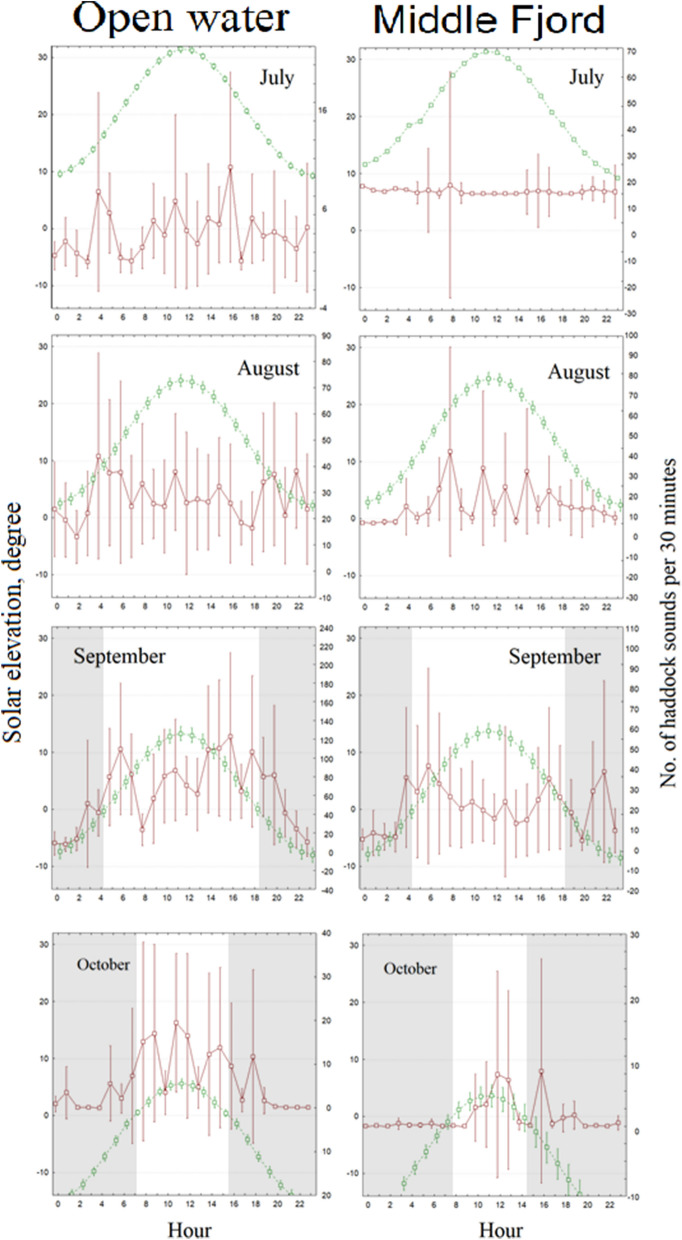
Mean number (whiskers: 0.95 confidential intervals) of haddock sounds per half hour (0–23) for each month of acoustic activity (brown line, right y scale) at the open-water and middle-fjord sites. The green line represents the solar elevation angle (left y scale). The shadow area indicates the dark hours, when the sun was below the horizon. Hour is expressed in UTC.

The mean number of haddock sounds per hour (0–23) for each month of acoustic activity (brown line, right y scale) at the open-water and middle-fjord sites is reported in Fig. 3. The green line represents the solar elevation angle (left y scale). The shadow area indicates the dark hours when the sun was below the horizon. Hour is expressed in UTC. The circadian pattern of haddock sounds changes over the months. In July and August, there are no evident vocalization peaks during the day alternation (see also Table 2). In open water, a circadian pattern is evident in September, when the alternation light–dark occurs (see the shadow area where negative values of solar elevation are present): two main peaks in acoustic activity were recorded during 4–6 am and 3–6 pm. Similarly, in the middle fjord, we found a peak during 4–6 am and another peak at nigth at approximately 10 pm. In October, the majority of acoustic activity was detected during the day.

**Table 2:** Results of the generalized additive models tested for haddock signals.

	Generalized additive models	Explanatory variables	Estimate	*X*^2^	*p*-Value	Deviance explained
Open water	July	DE-Tide	5.85	288.49	< 0.001	10.6%
		Hour	3.29	90.71	< 0.001	
	August	DE-Tide	5.97	1707.86	< 0.001	86.4%
		Hour	2.89	52.74	< 0.001	
	September	DE-Tide	7.95	1451.00	< 0.001	99.9%
		Hour	3.99	5570.00	< 0.001	
		Solar elevation	7.90	2621.00	< 0.001	
	October	DE-Tide	7.80	264.30	< 0.001	57.7%
		Hour	3.79	148.50	< 0.001	
		Solar elevation	7.78	229.10	< 0.001	
Middle fjord	August	DE-Tide	5.92	3371.30	< 0.001	32.2%
		Hour	3.97	682.90	< 0.001	
	September	DE-Tide	7.72	1370.00	< 0.001	39.1%
		Hour	3.99	6620.00	< 0.001	
		Solar elevation	7.98	22,042.00	< 0.001	
	October	DE-Tide	6.23	169.14	< 0.001	79.5%
		Hour	3.09	40.19	< 0.001	
		Solar elevation	7.39	81.33	< 0.001	

Hours, DE-Tide and Solar Elevation angles were considered as predictors of the model for September and October, while only Hours and DE-Tide for July and August.

**Figure 4:**
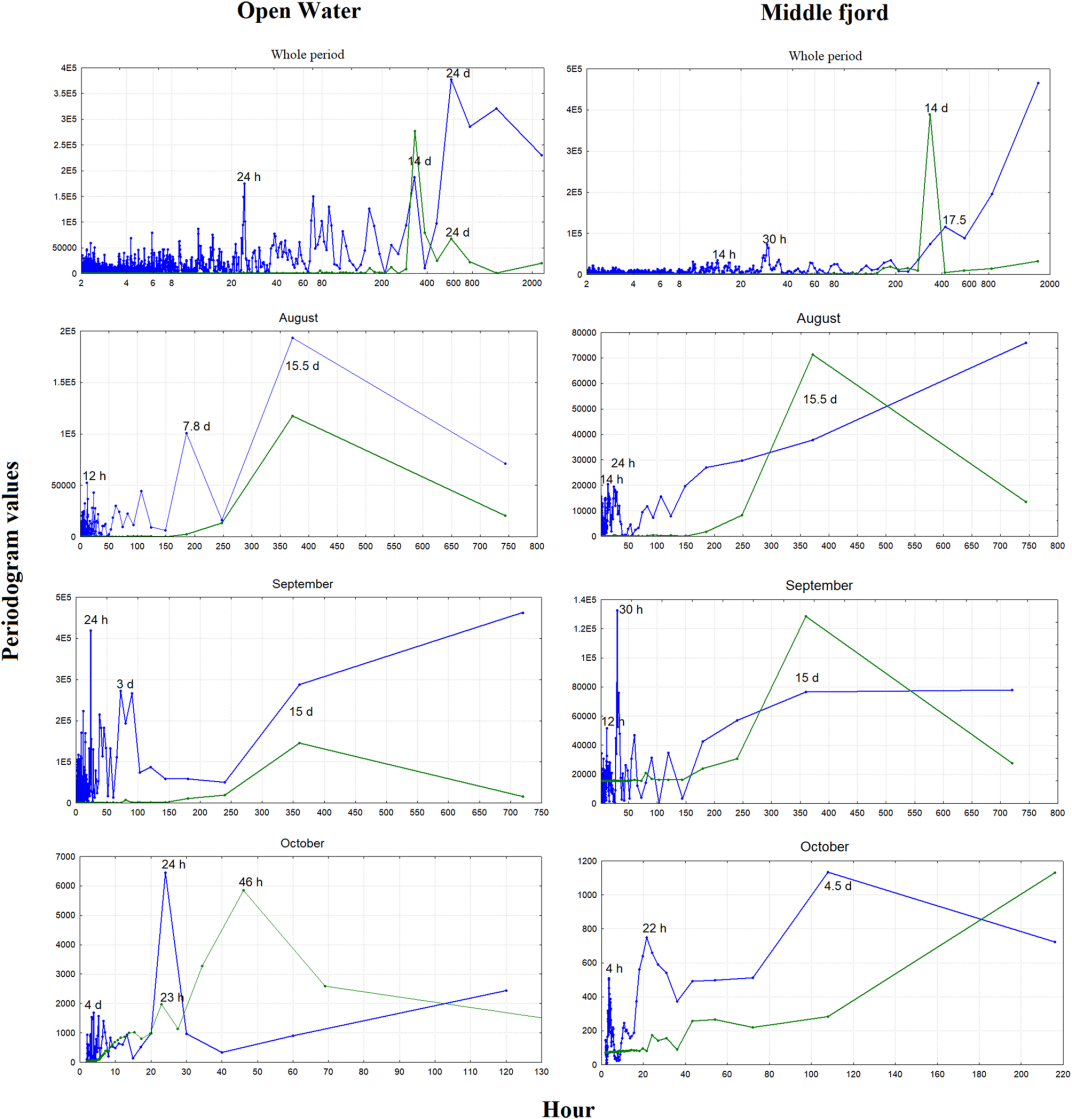
Periodograms, as showing the results of spectrum analysis, for haddock sound count (blue line) and DE-Tide (green line), considering the whole period (upper graphs) and each month of haddock acoustic activity (we did not consider July because the sounds occurred only on few days). The x-scale for the “whole period” graphs is expressed as a logarithm, whereas it is linear for the other, monthly graphs.

Figure 4 displays the periodograms, i.e., the results of the spectral analysis, for haddock sound count (blue line) and DE-Tide (green line), considering the whole period (upper graphs) and each month of haddock acoustic activity (we did not consider July because the sounds occurred only on a few days). The x-scale for the “whole period” graphs is expressed as a logarithm, whereas it is linear for the other, monthly graphs. Considering whole period of haddock acoustic activity, at the open-water site, we noted three main peaks: the first at 24 days, corresponding to the lunar day (and to the second peak in DE-Tide, green line); the second at 14 days, corresponding to the first peak in DE-Tide; and the third at a diel cycle of 24 h. In particular, if we look at each month separately, in August, the diel cycle is not evident, and the three main peaks are at 15.5 days, equivalent to the peak at DE-Tide at approximately 8 days and 12 h (equivalent to the tide period). Otherwise, during September and October, the first peak for both months occurs at 24 h.

In the whole period graph for the middle fjord (Fig. 4), small peaks at 17.5 days and at 30 h are observed. If we look at each month separately, in August, we do not see a clear pattern, and in September, we see clear peaks at 30 h and at 12 h (corresponding to the tide cycle); in October, we see peaks at 4.5 days and at 22 h, with a pattern that seems to follow the DE-Tide pattern.

**Figure 5:**
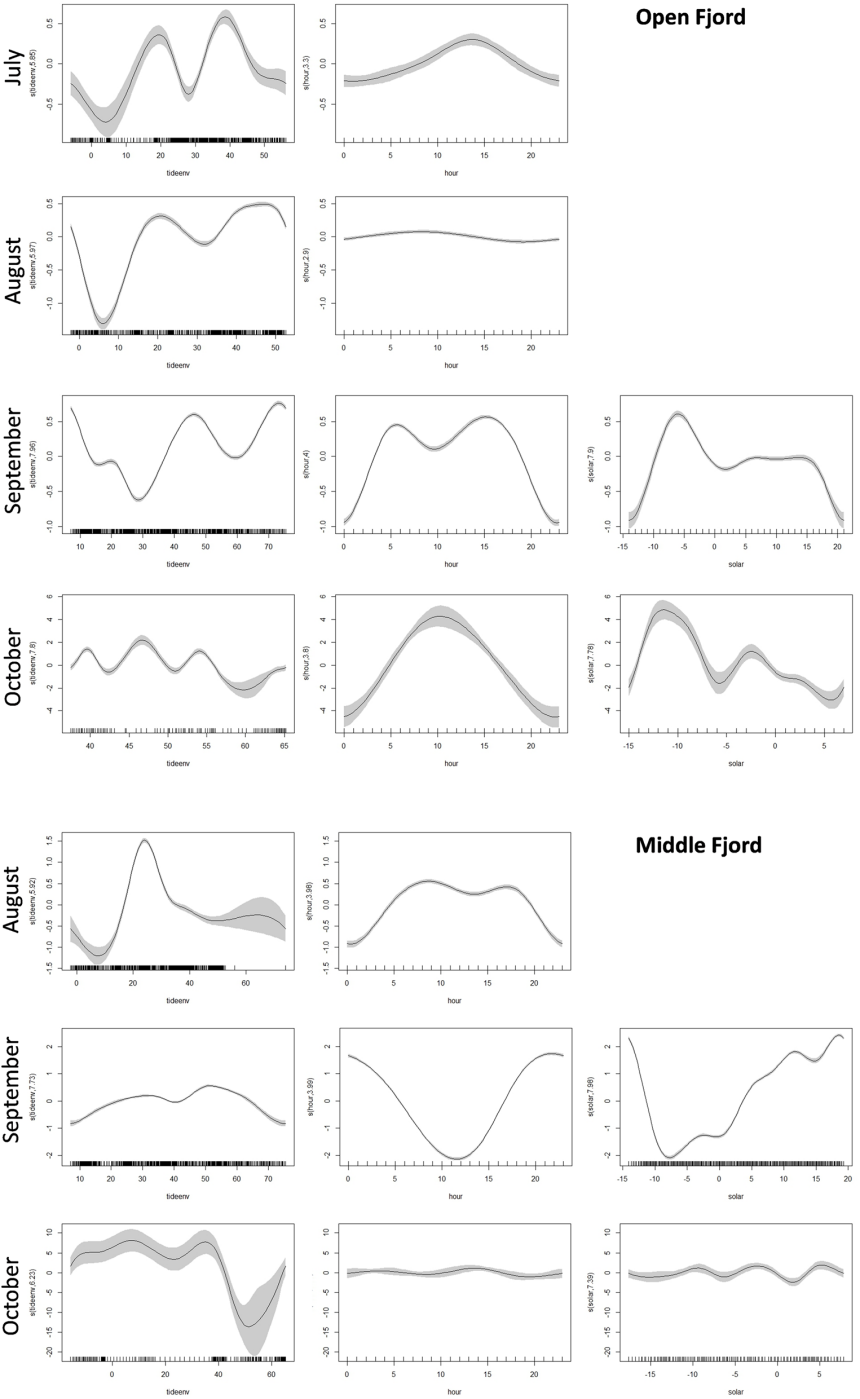
Response curves and number of haddock signals predicted by each of the variables in the zero-inflated generalized additive models (GAMs) at the open-fjord site in August and September (top) and in the middle-fjord site in August and September (below). The solid lines represent the smoothed estimates by the GAM, while the grey areas represent the approximate 95% confidence intervals. On the y axis the predicted number of haddock signals estimated by the model is displayed.

In the best-fitting GAM (Generalized Additive Models) (Table 2, Fig. 5), the predictor variables “Hours” and “DE-Tide” explained approximately 86% and 32% of the model deviance during August at the open-water and middle-fjord sites, respectively. These variables, together with the “Solar Elevation angles”, explained from 39% to almost 100% of the model deviance during September and October (Table 2, Fig. 5). Conversely, in July, only 10% of the deviance was explained by the predictor variables Hours and DE-Tide. As previously reported, the DE-Tide had the greatest effect at both sites until September, when the Solar Elevation angles and Hours variables revealed the influence of light–dark alternation.

During July and October, a smaller number of haddock sounds were detected in the recordings (see Fig. 1), and several zero values were present. Therefore, the results and reliability of the periodograms and GAM analysis during these two months may have been affected by these low numbers, while for August and September, the sounds were the most abundant.

### Haddock sound characteristics and spatial patterns

**Figure 6:**
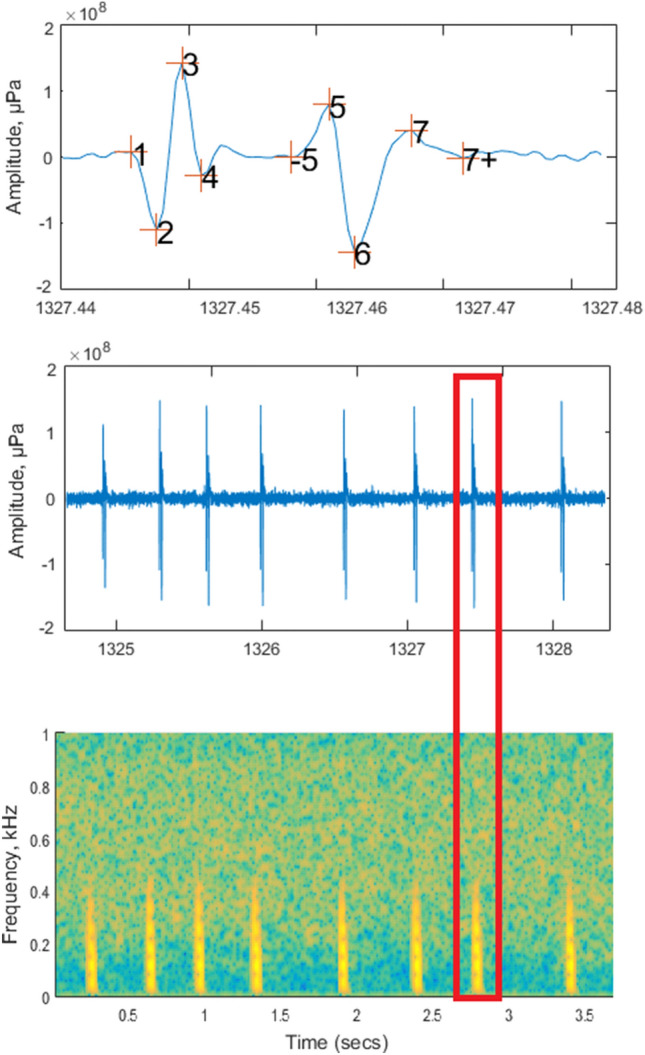
A multi-plot figure of the “check” MATLAB code. Upper: Selected waveform of a single sound unit, the knock, from a train. The numbers indicate the points at which time (t) and relative amplitude (a) measurements were taken. Middle: Waveform of the knock train (red rectangle is the selected sound). Lower: Spectrogram of the knock train (FFT length: 128, overlap: 100, sampling frequency 2000 Hz). This visualization was used by an operator to check the accuracy of the detection and the localization of the points from 1 to 7 + in the waveform.

The recorded sounds showed a clear double-pulsed low-frequency structure repeated in quick succession, similar to those recorded in the aquarium by Hawkins and Amorim (2000). Most of the recorded sounds were composed of short sequences (a few seconds) of irregularly spaced knocks (Fig. 6). No calls lasting for several minutes were recorded, and although the knock repetition rate was variable in the sounds, no knocks with a fast repetition rate merging to form a humming sound were detected.

**Figure 7:**
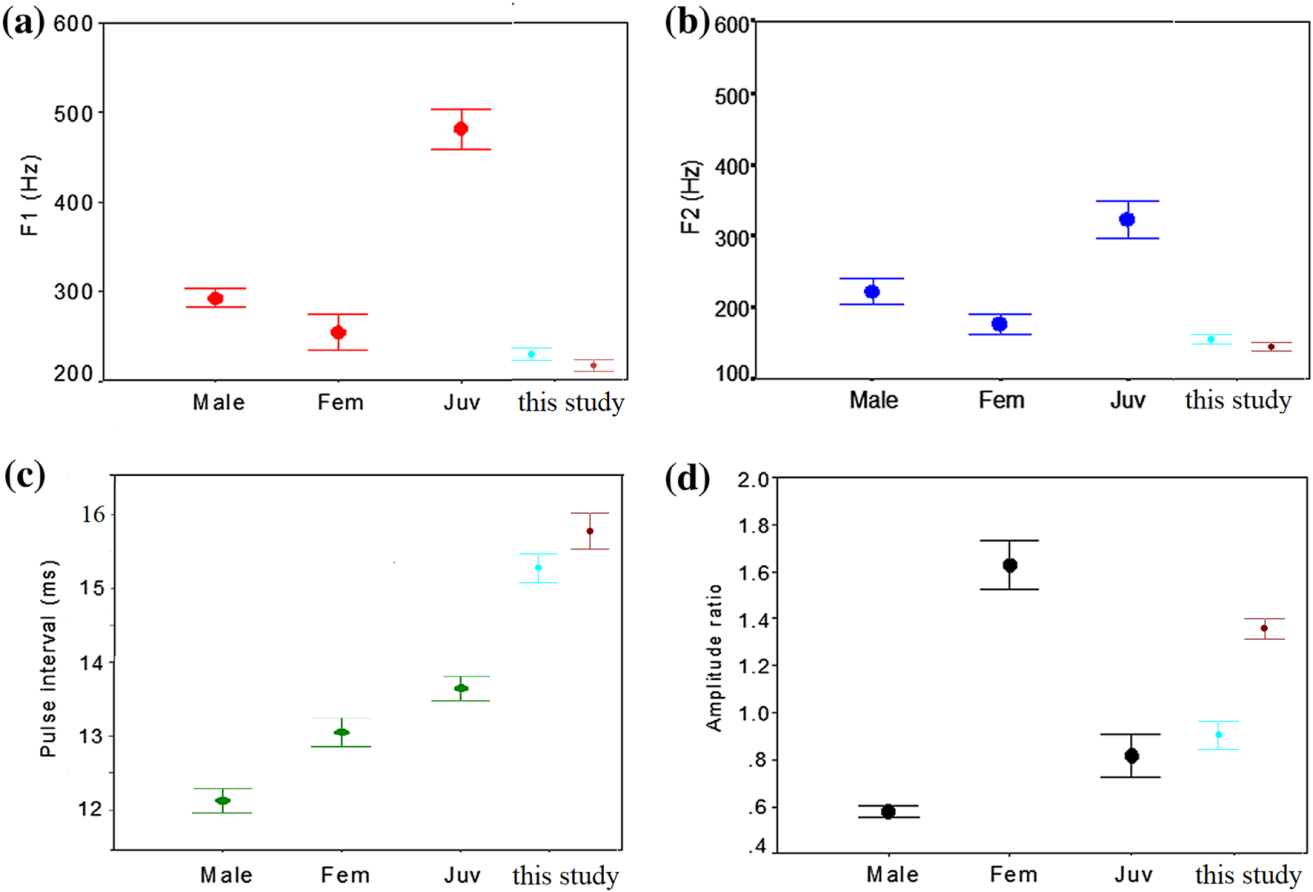
Acoustic parameters of haddock sounds recorded in an aquarium^12^ and in this study: frequency of the first pulse (F1) (**a**), frequency of the second pulse (F2) (**b**), pulse interval (**c**), and amplitude ratio (**d**). This study: Mean (error bar: 0.95 confidence interval) values obtained for the open-site (light blue) and middle-fjord site (brown). Modified from Casaretto et al. (2016).

A significant difference between sounds recorded at the open-water and middle-fjord sites was highlighted for F1 (i.e., the peak frequency of the first pulse of a knock¸ Mann–Whitney U test, p = 0.016) and for pulse interval and the amplitude ratio (Mann–Whitney U test, p < 0.05) but not for F2 (i.e., the peak frequency of the second pulse of a knock; Mann–Whitney U test, p = 0.071) or sound duration (Mann–Whitney U test, p = 0.149). The mean values (with 0.95 confidential intervals) of the considered parameters are reported in Fig. 7 for comparison with the mean values (with 0.95 confidential intervals) for haddock males, females and juveniles reported by Casaretto et al. (2016). Knocks acoustic measurements are reported for both sites and separately for the two sites in Table 3.

**Table 3:** Acoustics characteristics of haddock sounds.

Site	Acoustic measure	Mean	Std. Dev	Median	10th percentile	90th percentile	Min	Max
Middle-fjord and open-sea (no = 451)	F_1_, Hz	229.9	40.1	222.2	173.9	285.7	125.0	363.6
	F_2_, Hz	149.8	30.8	142.9	117.6	181.8	88.9	333.3
	Peak frequency, Hz	129.6	39.2	121.0	101.0	189.0	42.0	269.0
	Duration, ms	21.4	1.6	21.3	19.5	23.5	17.2	28.4
	Amplitude ratio	1.2	0.3	1.2	0.6	1.6	0.4	2.5
	Pulse interval, s	15.6	1.3	15.4	14.3	17.3	12.4	20.3
Middle-fjord (no = 238)	F_1_, Hz	225.5	39.5	222.2	173.9	285.7	125.0	307.7
	F_2_, Hz	146.7	27.9	142.9	114.3	173.9	88.9	333.3
	Peak frequency, Hz	131.0	33.1	121.5	106.0	186.0	43.0	269.0
	Duration, ms	21.7	1.8	21.3	19.8	24.1	18.5	28.4
	Amplitude ratio	1.4	0.3	1.3	1.1	1.7	0.9	2.5
	Pulse interval, s	15.8	1.43	15.4	14.5	18.1	12.4	20.3
Open-sea (no = 213)	F_1_, Hz	234.8	40.3	235.3	181.8	285.7	142.9	363.6
	F_2_, Hz	153.2	33.4	148.1	117.6	190.5	97.6	333.3
	Peak frequency, Hz	128.1	45.1	121.0	53.0	193.0	42.0	252.0
	Duration, ms	21.2	1.4	21.3	19.1	22.9	17.2	25.5
	Amplitude ratio	0.9	0.3	0.9	0.6	1.3	0.4	2.5
	Pulse interval, s	15.3	1.1	15.3	13.9	16.6	12.7	19.1

### Haddock sound characteristics and their possible relations to population structure

Table 4 presents the distributions of sounds at the two monitored sites according to the amplitude ratio between the two pulses that form a haddock sound. This ratio was suggested to be indicative of the sex and age of the emitting fish in the above mentioned aquarium study. As a result, among the 451 recorded sounds selected, 44% could be attributed to juvenile fish and 41% to females, whereas only 15% presented the typical characteristics of sounds emitted by mature haddock males. Table 5 compares the sound characteristics of the different emitter classes defined based on the amplitude ratio. The peak frequency of the first pulse of a knock (F1) is significantly different among the three groups, i.e., slightly higher in male-like sounds than in female-like sounds, consisten with the finding of the previous aquarium study. Nevertheless, the F1 of the juvenile-like sounds recorded in nature appears to be far lower than expected. No differences were detected for the peak frequency of the second pulse of a knock (F2), whereas a statistically significant difference in the pulse interval was found among the three groups: female-like sounds showed a longer pulse interval than male-like sounds, in accordance with the findings of Casaretto et al. (2016). Those authors, however, reported an average pulse interval shorter than those indicated here. Again, the pulse intervals of the juvenile-like sounds were longer than expected according tothe abovementioned laboratory study.

**Table 4:** Distribution of haddock sounds according with the sound amplitude ratio (male-like sounds refer to an amplitude ratio < 0.8; juvenile-like sounds refer to 0.8 < amplitude ratio < 1.1; female-like sounds refer to an amplitude ratio > 1.2).

	Open-site		Middle-fjord site		Both sites	
	N. sounds	Percentage	N. sounds	Percentage	N. sounds	Percentage
Female-like sounds	24	11.2%	161	67.6%	185	41.0%
Male-like sounds	69	32.4%	0	0	69	15.3%
Juvenile-like sounds	120	56.4%	77	32.4%	197	43.7%
total	213	100%	238	100%	451	100%

**Table 5:** Characteristics (Mean ± Stand. Dev) of the knocks recorded in the Arctic fjord Kongsfjorden, divided according with the sex of the emitter (F1 refers to the peak frequency of the first pulse of a knock; F2 refers to the peak frequency of the second pulse of a knock; the pulse interval is the interval between the start of the first pulse and the start of the second pulse).

	Female-like sounds	Male-like sounds	Juvenile-like sounds	Statistic significance
F1	216 ± 40 Hz	250 ± 30 Hz	235 ± 38 Hz	P < 0.001
F2	146 ± 27 Hz	150 ± 35.8 Hz	152 ± 32 Hz	P = 0.17
Pulse interval	16 ± 1 ms	15 ± 0.9 ms	15 ± 0.9 ms	P < 0.001

Statistical significant differences were tested at the 0.05 probability level using a non-parametric test (Kruskal–Wallis Test).

**Figure 8:**
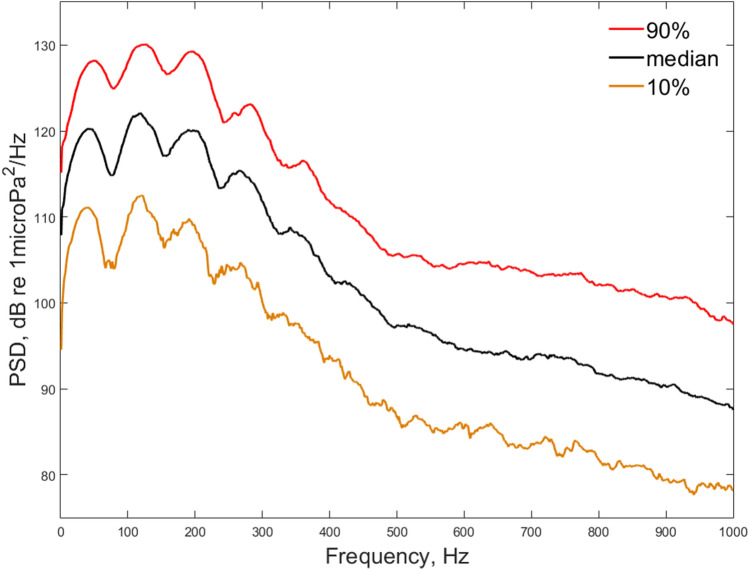
Median (10th–90th percentile) power spectral density (PSD, dB re 1 μPa2/Hz) for all selected haddock sounds (no = 451). PSD was calculated in the time window (see Fig. 6) between the start (t1) and the end of knock (t7 +).

The median power spectral density of selected sounds (no = 451) is shown in Fig. 8. The PSD showed different peaks between 50 and 400 Hz, with the highest at approximately 130 Hz (see also Table 3).

## Discussion

The recorded sounds confirmed the presence of haddock in the fjord due to their consistence with the sounds recorded in the laboratory. This result is consistent with that of a precevious study based on trawling and fishing gear^31^.

Although highly likely produced by haddock, the recorded sounds showed some variance compared to those in studies performed in captivity^12^, presenting lower peak frequencies and a longer pulse interval (Fig. 7). This could be explained by an effect of water temperature on the fish neuromotor system, as the water temperature within the aquarium tank was approximately 8 °C, whereas the internal sensors of the recorders detected sea temperatures between + 4 and − 2 °C for all the sampling periods. The effect of temperature on fish vocalizations has been demonstrated in many species^32–34^, showing that temperature increases are associated with longer pulses, shorter pulse intervals and higher frequencies of emitted sounds. On the other hand, the sound peak frequency of approximately 130 Hz reported here is consistent with the peak frequency lower than 200 Hz reported by Stanley et al.^35^ for haddock calls recorded in the sea during the winter spawning period.

### Temporal pattern

Our analysis revelead that haddock sounds are present in Kongsfjorden from July to October, i.e., outside the spawning period of this species. The temporal pattern at the open-water site, where we recorded the most haddock sounds, showed a gradient shift from long periodicity linked to the moon tide to shorter periodicity directly correlated with sunlight (see Fig. 4 and Table 2). At the middle-fjord site, we did not detect these clear temporal patterns. This is probably related to the significant reduction in haddock sound abundance and the complete lack of soundson many days. The results presented here showed that the periodicity of haddock sounds follows the most important amplitude oscillation of physical forcing available in the environment. First (Table 2), during July August, DE-Tide is the strongest physical oscillation, and then, during September–October, light/dark alternation (with a 24-h period) is initiated (see also Fig. 4). Although this result is based only on vocal production, it suggests that the haddock activity rhythms persist during the polar day, as in the case of the burbot (*Lota lota*)^25,26^. This indicates adaptive plasticity in timing cues for acoustic activity in *Melanogrammus aeglefinus*, possibly due to the recent presence of this species in the polar area or perhaps the cycle of its prey. According to Reneaud et al.^28^, the diets of co-occurring juvenile haddocks in fjords, open water, and sea ice around Svalbard are mainly based on zooplankton, i.e., appendicularians and *Limacina* spp., and the activity rhythms of the zooplankton have been documented in the polar area. for example, a recent study showed that during the polar night, vertical zooplankton migrations are driven by moonlight in synchrony with the altitude and phase of the moon, with a shift from solar-day to lunar-day vertical migration^36^.

In Kongsfjorden acoustic rhythms have also been reported also for the bearded seal *Erignathus barbatus,* whose vocalisation rate varies significantly both daily and monthly^37^, showing a correlation with solar elevation when light and darkness turnover is occurs.

### Spatial pattern

Haddock sounds in the Arctic fjord Kongsfjorden were not homogeneously distributed at the three sites of recordings, and were characterized by an internal–external gradient corresponding to the gradient of Arctic–Atlantic oceanographic conditions. No vocalizations have been detected in the inner part of the fjord (glacier site), i.e., the most “Arctic” part, which is affected by the colder, fresh water coming from the run-off of glaciers and ice calving^38^. In contrast, the highest density of haddock sounds was recorded in the most “Atlantic” part of the fjord, the open-water site, which is influenced by water masses from the Fraim Strait belonging to the West Spitsbergen Current^39^. In the middle part of the fjord, which is probably characterized by conditions intermediate to those of the Arctic and Atlantic parts, the number of recorded sounds was significantly lower than that at the open-water site. This can be explained by the haddock being an Atlantic species that usually adapts well to temperatures up to 10 °C, although 0-group haddock fish (fish in the first year of their life) have been recorded above 80° N^40^ and a haddock spawning area has been reported in the Norwegian Sea between 60° and 75° N^1^. An effect of water depth on the distribution of fish (sounds have been recorded at 75 m at the open-water site and at 20 m at the middle-fjord site) cannot be excluded. However, the recording sites had different oceanographic conditions, and one, the middle-fjord site, presented la shallower depth (20 m versus 75 m for the other two sites). These differences, especially in depth, could affect detection among our recording sites due to differences in acoustic propagation among the sites. The detection range of a certain sound by a recorder depends on multiple factors, such as the sound source level (unknown for haddock sounds), background noise, bathymetry, hydrophone sensitivity, and software detector sensitivity. For this reason, in the future, the source level of haddock sounds should be assessed, and further studies in the natural environment should consider the detection range of the monitoring system to clarify the sampling volume at each recording site.

### Haddock behaviour and population characteristics inferred by their sounds

Bacause they were recorded outside the reproductive season, the vocalizations reported here are not related to the breeding behaviour of the haddock. This is supported by the sound types, which lack the typical acoustic pattern associated with the patrolling behaviour (i.e., knocks repeated in trains at intervals of between 140 and 50 ms for minutes) or continuous hums (i.e., rapidly repeated knocks merging to form a humming sound) that are produced during reproduction, as described in the captivity studies^8–10^ as well as recorded at sea^6,35^. As a consequence, we can conclude that the species is not using the Arctic fjord Kongsfjorden for spawning.

For the first time, we applied sound analysis based on captivity studies, showing that the vocalizations recorded in the fjord are not mainly produced by mature haddock males. This conclusion is based on using the sound amplitude ratio as the most reliable variable for distinguishing the sex of the emitting fish: in the laboratory recordings, the amplitude ratio of females was always equal to or greater than 1, whereas it never exceeded 1 in males’ sound units. This sex difference was accompanied by sexual dimorphism in haddock drumming muscle mass, similar to that found in other gadiids: haddock males were found to have more well-developed drumming muscles than females of similar size throughout the whole year^12^. The relationships between physiological parameters and the resultant sound waveform were further described by Casaretto et al. (2016)^12^. On the other hand, clear overlap between males and females was evident here for the frequencies of the first (F1) and second (F2) pulses as well as for the pulse interval. These findings exclude a role of these sound features in sex recognition.

In the present study, the amplitude ratio was slightly less efficient in distinguishing haddock fish maturity. According to the captivity study^12^, the sound units produced by immature fish consist of two pulses with similar amplitudes (i.e., with an amplitude ratio approximately 1) and present higher peak frequencies than those of adults. The sounds recorded at sea, however, do not show clear intra-population differences in frequency values (Fig. 7); therefore, there is a level of uncertainty. In captivity, the fish sound parameters may have been affected by the recording conditions due to distortion, reverberations and reflections inside the fibreglass stock tanks^12,41^ where juveniles were located and maintained. At sea, the size (unknown) of the juvenile callers may have played a role in the recorded sound structure. Poor development of the drumming muscle has been proven in haddock juveniles^10,11^, as in other juvenile stages of gadiids^39^; however, our knowledge about the ontogeny of fish sound production is poor and thus far limited to a few fish species^38^, so the effect of fish size on sound structure is difficult to fully evaluate. In any case, if we consider the aquarium values for the amplitude ratio of juvenile sounds (0.6 ± 0.2 SD for males and 0.8 ± 0.3 SD for juveniles) and our classification (male-like sounds have an amplitude ratio < 0.8; juvenile-like sounds: 0.8 < amplitude ratio < 1.1), it seems more plausible that a juvenile sound would be included in the ‘male-like’ category than a male sound would be included in the ‘juvenile-like’ category. This supports our analysis.

We conclude that the fjord is populated mainly by juvenile haddock. Further, the two recording sites seem to differ in their population abundance and composition, with predominantly females at the middle-fjord site and juveniles at the open-water site. These conclusions must be made while taking into account the technique used to obtain them. In passive acoustic monitoring, the sampled volume is limited to the detection range of the recorder system, which in this study could change with oceanographic conditions of the two sites. However, our results are in agreement with those of research on the Ichthyofauna of Kongsfjorden that quantitatively described the local shallow-water fish community^31^ captured using trammel and fyke nets and showed that only juvenile haddock (standard length: 6–19 cm; moda: 17.5 cm) were present. Further studies are needed to fully confirm the results of our analysis.

Passive acoustic monitoring (PAM) is a valid alternative to more expensive and logistically difficult techniques for monitoring the distribution of “new invasive species” in an area that is difficult to study, such asthe Arctic. This is also valid outside the reproductive season, as demonstrated by this work. Long-term PAM can provide important information on the relationship between soniferous species present in Svalbard (for example, cod, haddock and polar cod) and their new adaptations to the different light/dark cycles in this changing environment as well as information on the sex and maturity of the individuals present.

## Materials and methods

### Study area

**Figure 9:**
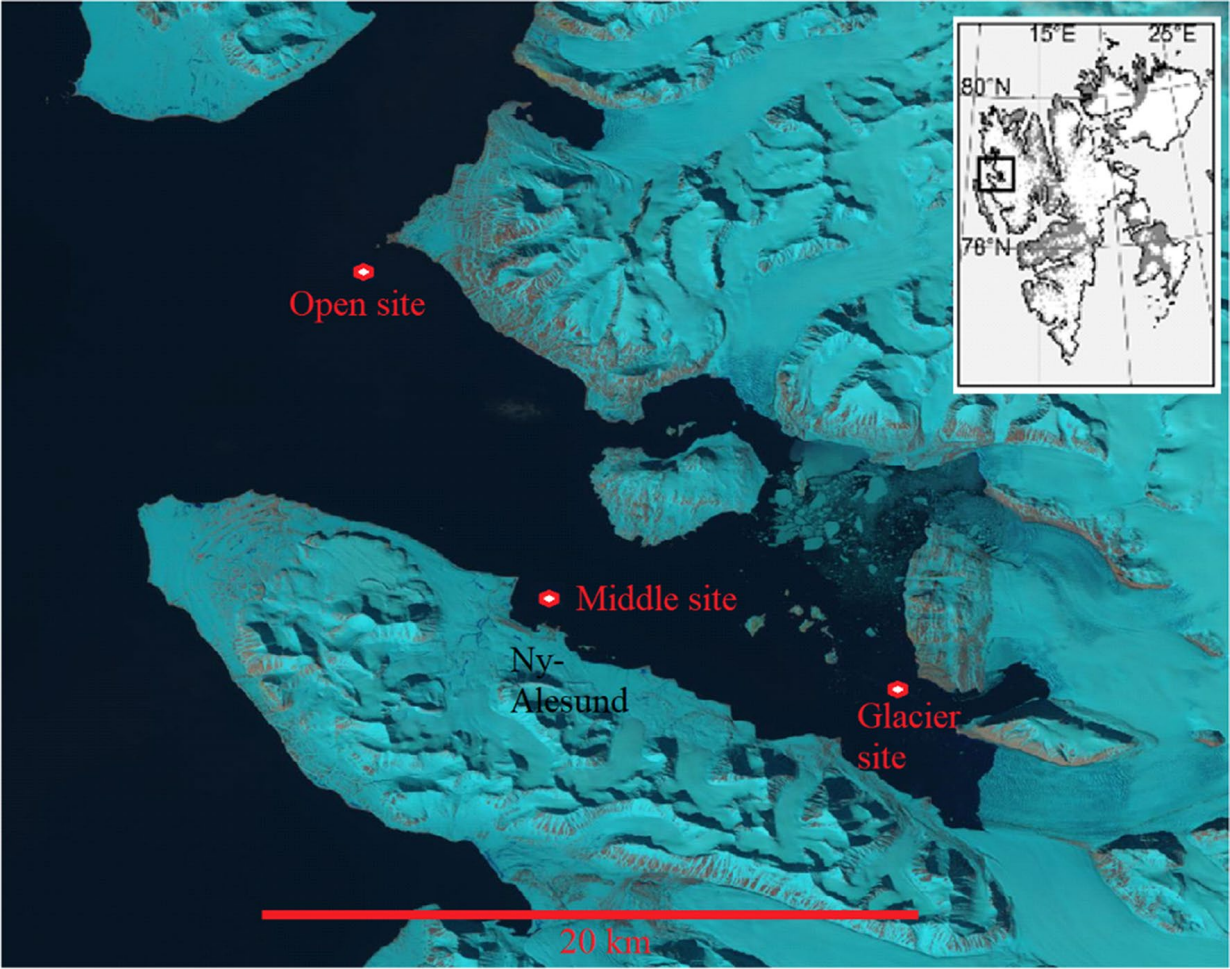
Study area: in the insert, the Svalbard Islands are represented, while in the principal image, Kongsfjorden, Western Svalbard, with the position of the recorder sites (red dots). (Credit: U.S. Geological Survey, Department of the Interior/USGS).

The study area is located within Kongsfjorden (Spitsbergen, Svalbard Archipelago, Norway; Fig. 9) at 78°–79° North and 11°–12° East. The fjord oceanography is regulated by the mixing of melting ice, cold Arctic waters, and warmer Atlantic water masses^42^. Actively calving marine-terminating glaciers supply icebergs and freshwater input, which, together with other physical factors, determine the local species composition^39^. Glacier fronts are upwelling areas for zooplankton^43^ and attract many top predators. However, climate-induced changes in oceanographic conditions are significantly altering Kongsfjorden habitats^39^. Tidewater glaciers in Kongsfjorden are retreating^44^, and recently, during the wintertime, sea ice formed only in the inmost parts of the fjord (Fig. 9).

Kongsfjorden, located at 79° N and 12° E along the western coast of Spitsbergen Island is a glacial fjord. Here, however, colder Arctic water masses mix with warmer Atlantic waters belonging to the West Spitsbergen Current^39^; therefore, a steep environmental Arctic-Atlantic gradient is generated from the inmost part of the fjord to its mouth^38^. This gradient, with short- and long-term changes in intensity, could influence the benthic and pelagic species of the fjord and result in a mixture of Atlantic and Arctic species in close association. In the last decade, Kongsfjorden has shifted towards having Atlantic features^30^. Although the haddock distribution area includes the Svalbard Archipelago, Kongsfjorden represents the northernmost limit of its distribution^40,45^. A study of fish species composition and abundance in the shallow waters of Kongsfjorden^31^ revealed the presence of juvenile haddocks (0.4% abundance with respect to all fish species) with standard lengths between 6.0 and 19.0 cm (mode: 17.5 cm).

### Acoustic data collection

Data were collected year-round from April 2014 to March 2015 by using three autonomous passive acoustic recorders (SM2, Wildlife Acoustics, US). The instruments were deployed at three different sites (Fig. 9). The first (hereinafter called glacier site) was located in the inner part of Kongsfjorden (78°54.74′ N–12°24.31′ E), at a distance of approximately 4 km from the Kronebreen glacier front, one of the largest fast-flowing and actively calving marine-terminating glacier streams of the Svalbard Islands; the second (hereinafter called middle-fjord site) was located in the middle part of Kongsfjorden (78°55.93′ N–11°54.65′ E), at a distance of 15 km from the Kronebreen glacier; and the third (hereinafter called open-water site) was located at the mouth of the fjord (79°03.22′ N–11°32.83′ E), 27 km away from the Kronebreen glacier.

The hydrophones had a recording bandwidth of 8 Hz–150 kHz and a sensitivity of − 165 ± 4 dB re 1 V/μPa in the 25 Hz–10 kHz band. The recorders were located at a depth of 75 m for the glacier and open-water sites, and of 20 m for the middle-fjord site; they were 8 m from the bottom, anchored with a 35-kg ballast and kept vertical by a small subsurface buoy. Furthermore, to avoid any noise due to moving parts, the connections between the recorders, the acoustic release and the ballast, as well as those with the buoy, were composed of non-metallic ropes.

We set the sampling frequency at 48 kHz with a resolution of 16 bits, and no pre-amplification or filtering was applied (except for the antialiasing filter automatically applied by the recorder) during the recordings. Depending on the period of the year and the availability of logistic support at different sites, data were collected following different schedules with a daily duty cycle of 100% (all days) or 30% (one day every three days). For each day sampled, we used a duty cycle of 50% (the first 30 min of every hour) (Table 1). The recorders were recovered for maintenance every 4 months to change batteries and clear the storage memory.

### Acoustic data analysis

All data, resampled at 2 kHz or 8 kHz and filtered with a high-pass filter (cut-off frequency: 40 Hz), were analysed using two sets of Matlab code developed with the aim of detecting and characterizing all the haddock calls. The first code used the Teager Kaiser operator^46^ and the function “findpeaks” to amplify and select the probable pulses from haddock in the files resampled at 2 kHz. An empirical threshold, always the same for all the analyses, was adopted to select all the pulses that exceeded this threshold. Then, all these selected pulses were visualized using their oscillograms and spectrograms (see Fig. 6) to check whether they were actual haddock pulses.

To achieve accurate measurements, a total of 451 sounds with a good signal/noise ratio and with a uniform temporal distribution (to uniformly represent all hours of the day and each week of the month) were used to analyse the sound structure of the recorded knocks, with 213 recorded at the middle-fjord site and 238 recorded at the open-water site. No haddock sounds were recorded at the glacier site. To avoid strong dependency of the measurements from a few individuals, for each file, we used a maximum of 10 signals.

Following the studies of Casaretto et al.^9,12^, a second set of MATLAB code was used on the files resampled at 8 kHz to measure and check by an operator the temporal position and the correspondence amplitude of the peaks of the two pulses that composed each knock (Fig. 6). From the signals, we measured the following acoustic parameters: (1) pulse duration: total duration of the double pulse measured as the temporal difference between points t7 and t1; (2) the frequency of the first pulse of a knock, calculated as f_1_ = 0.5(t3 − t2)^−1^; (3) the frequency of the second pulse of a knock, calculated as f_2_ = 0.5(t7 − t6)^−1^; (4) difference in amplitude between the first and second pulses, calculated as (a7 − a6) (a3 − a2)^−1^; and (5) the pulse interval, i.e., the interval between the start of the first pulse and the start of the second, calculated as t6 − t2.

Differences in pulse amplitude can be considered indicative of the sex and maturity of the emitting fish. Following Casaretto et al. (2016)^12^, (1) in male sounds, the first pulse of the knock is higher in amplitude than the second pulse, resulting in an amplitude ratio value lower than 1 (0.6 ± 0.2 SD); (2) females produce sound units composed of two pulses, with the second pulse higher in amplitude than the first, resulting in an amplitude ratio value higher than 1 (1.6 ± 0.6 SD); and (3) immature fish show two pulses with similar amplitudes but different polarities, thus resulting in an amplitude ratio close to 1 (0.8 ± 0.3 SD). Accordingly, all the sounds with an amplitude ratio lower than 0.8 were assumed to be produced by haddock males (male-like sounds), sounds with an amplitude ratio ranging between 0.8 and 1.1 were assumed to be produced by juveniles (juvenile-like sounds), and all the sounds with an amplitude ratio higher than 1.2 were assumed to be produced by haddock females (female-like sounds).

To evaluate any differences in these acoustic parameters between sites, the non-parametric Mann–Whitney U test was used.

For selected signals (no = 451), the power spectral density (PSD) (dB re 1 μPa^2^/Hz) was calculated with Welch’s overlapped segment averaging estimator method (Fig. 8). The calculation was performed in the time window between t7 + and t1 (see Fig. 6). Then, we calculated the frequency peak (Hz) as the frequency corresponding to the highest amplitude in the power spectral density.

### Temporal pattern: relations with the diel cycle of light and tide

Tide tables for Kongsfjorden were obtained online (https://www.kartverket.no/). The down envelope tide (DE-Tide) was obtained using the MATLAB function Envelope (designed by L. Wang). DE-Tide represents the oscillation of tide amplitude (in this case, the lower amplitude, or neap tide). This oscillation occurs every 15 days, when the sun and the moon form a right angles.

The position of the sun in the sky was taken into account by using solar elevation angles with respect to the horizon line. Positive and negative values of the solar elevation angles indicate the presence and absence of solar radiation, respectively, while the absolute value is a proxy of the distance from the horizon. The solar elevation angles were obtained as output from the MATLAAB function “SolarAzEl” (programmed by D. C. Koblick, 2013) after inputting the geographical coordinates, metres above sea level (i.e., 0 m) and UTC time.

To explore temporal patterns in the presence and abundance of haddock sounds, we applied single-series spectrum analysis, considering only the period of occurrence of haddock sounds at each recording site.

To evaluate differences in the abundance of haddock sounds between sites, the non-parametric Mann–Whitney U test was applied to all data set, considering each month of acoustic activity separately.

Moreover, generalized additive models (GAM) (R “mgcv” package version 1.8–28; Wood^47^) were used to evaluate which predictors best explained the occurrence of haddock sounds, which were considered the response variable, over the months. Signals were tested as a function of the predictors hours (both daytime and nighttime, discrete variable ranging from 0 to 23), DE-Tide, and Solar Elevation angles as described above for each month, at both the open-water and middle-fjord sites. A zero-inflated Poisson regression distribution and an identity link function were chosen. Cyclic cubic regression splines were used for all the explanatory variables. All the analyses were performed in R^48^.
